# 

**Published:** 1901-08-24

**Authors:** 


### NOTICE TO CORRESPONDENTS.

All MSS., Letters, Books for Review, and other matters intended for the Editor should be addressed The Editor, "The Hospital," The Hospital Building, 28 & 29 Southampton Street, Strand, W.C.
The Editor cannot undertake to return rejected MS., even when accompanied by stamped directed envelope.

**Notice to Advertisers and Subscribers.**

All Advertisements, Orders for Copies of The Hospital, and Business Communications should be addressed to **THE MANAGER (Not to the Editor),** The Hospital, 28 & 29 Southampton Street, Strand, London, W.C.

The Cost of Subscription, post free, is as follows:—Per week, 2½d.; per quarter, 2s. 8d.; per half-year, 5s. 3d.; per annum, 10s. 6d. Foreign:—Per annum, 15s. 2d., payable, in all cases, in advance.

**NOTICE.—Vol. XXIX. of "The Hospital" (October 1900, to March, 1901), in handsome cloth binding is now on sale at the Office of this Journal, price, 7s. 6d. Cases for binding the Numbers contained in this Volume 1s. 6d. Index to Vol. XXIX., 2½d., post free.**

**The Monthly Part for September will be ready on September 2. Price 6d., by post 8d.**

### SCRAPS AND GLEANINGS.

SINCE 1891, when the Surrey Convalescent Home, Seaford, was founded, over 5,000 patients have resided at the institution. During the past year the finances of the home have not flourished, and this year's accounts show a deficit of £135.

A SCHEME is on foot, inaugurated by a Viennese lady, to open a Jewish Nursing Home in Vienna, where Jewish girls are to be trained as nurses. This innovation is due to the desirability of having Jewish nurses to attend the patients in the Jewish hospital which has been established in Vienna; £1,000 with the funds in hand is now wanted to start this nurses' home.

ON the invitation of Lord Duncannon (the chairman) and the directors of Bovril, Limited, the members of the British Congress on Tuberculosis visited the laboratories and factory of Bovril, Limited. Samples of the rations as supplied to the Nansen, Jackson-Harmsworth, and Wellman Polar Expeditions were on view, whilst an interesting feature was an exhibit of samples of goods supplied for the Royal Antarctic Expedition which has just started.

THE sixty-fourth annual report of the Cardiff Infirmary, just issued, states that the financial position of the institution is in a much better state than the committee have had to record for several years past. Grateful recognition is accorded to the efforts of the Mayor of Cardiff and the editor of the *Western Mail*, who both started special funds on behalf of the institution. These special funds amounted to £12,568 odd, £5,505 of which came through the *Western Mail* fund.

### THE STUDENT OF MEDICINE.

### APPOINTMENTS.

**H. R. Beale,** M.R.C.S., L.R.C.P., appointed Assistant Resident Medical Officer to the Leeds General Infirmary. **J. H. Bendle,** M.B., L.R.C.P.Lond.. M.R.C.S.Eng., appointed District Medical Officer of the Uttoxeter Union. **E. N. Berryman,** M.A.Oxon., M.R.C.S., L.R.C.P.Lond., appointed District and Workhouse Medical Officer of the Llandovery Union. **S. J. Brooks,** M.R.C.S.Eng., appointed District Medical Officer to the Tiverton and St. Thomas's Unions. **R. J. Coulter,** M.B., F.R.C.S.I., appointed Ophthalmic Surgeon to the Newport and Monmouthshire Hospital. **H. J. Curtis,** B.S.Lond., F.R.C.S.Eng., appointed Assistant Surgeon to the Royal Hospital for Children and Women, Waterloo Road. **O. Gruner,** M.R C.S., L.R.C.P., appointed House Physician to the Leeds General Infirmary. **H. Hollis,** M.D.Camb., B.C., appointed District Medical Officer and Medical Officer of the Workhouse of the Wellingborough Union. **Vincent Howard,** M.R.C.S., L.R.C.P., appointed Medical Officer of the Union Workhouse, Buckingham, and Medical Officer and Public Vaccinator for the Leckhampstead District of the Buckingham Union. **H. R. Jacobs,** L.R.C.P.Edin., M.R C S.Eng., appointed Medical Officer for the Sulgrave District of the Brackley Union. **A. J. Kennedy,** L.S.A., appointed Medical Officer for the Fulstone District of the Huddersfield Union. **Thomas Hillhouse Livingstone,** M.B., Ch.B.Edin., appointed District Medical Officer and Medical Officer to the Workhouse, and also Public Vaccinator in the Stanhope District of the Weardale Union. **A. E. Mahood,** M.B., M.Ch.R.U.I., appointed Medical Officer of Health for the Northam Urban District. **T. C. Mitchell, jun.,** M.R.C.S., L.R.C.P.Lond., appointed District Medical Officer of the Thirsk Union. **T. C. Oxford,** M.R.C.S., L.R.C.P.Lond., appointed Assistant Medical Officer to the Salford Union Infirmary. **E. E.**

Parrett, M.R.C.S.Eng., L.R.C.P.Lond., appointed Assistant Medical Officer of the Brentford Union Infirmary, Workhouse, and Schools. **A. L. Perkins**, L.R.C.P., L.R.C.S.Edin., appointed District Medical Officer of the Swansea Union. **G. H. Ransome**, M.R.C.S., L.R.C.P.Lond., appointed District Medical Officer of the Depwade, Loddon, and Clavering and Wangford Unions. **W. E. Robinson**, B.A.Oxon., M.R.C.S., L.R.C.P.Lond., appointed District Medical Officer of the Wokingham Union. **W. F. Stevenson**, L.R.C.P., L.R.S.Edin., appointed District Medical Officer of the Henley Union. **M. Stewart**, L.R.C.P., L.R.C.S.Edin., appointed Medical Officer for the Wigton District of the Wigton Union. **J. Watson**, L.R.C.P., L.R.C.S.Edin., appointed District Medical Officer of the Alnwick and Glendale Unions. **A. G. Wilkins**, M.B., B.Ch.Vict., appointed Assistant Medical Officer to the Salford Union Infirmary.

